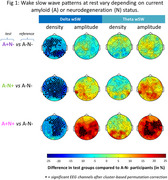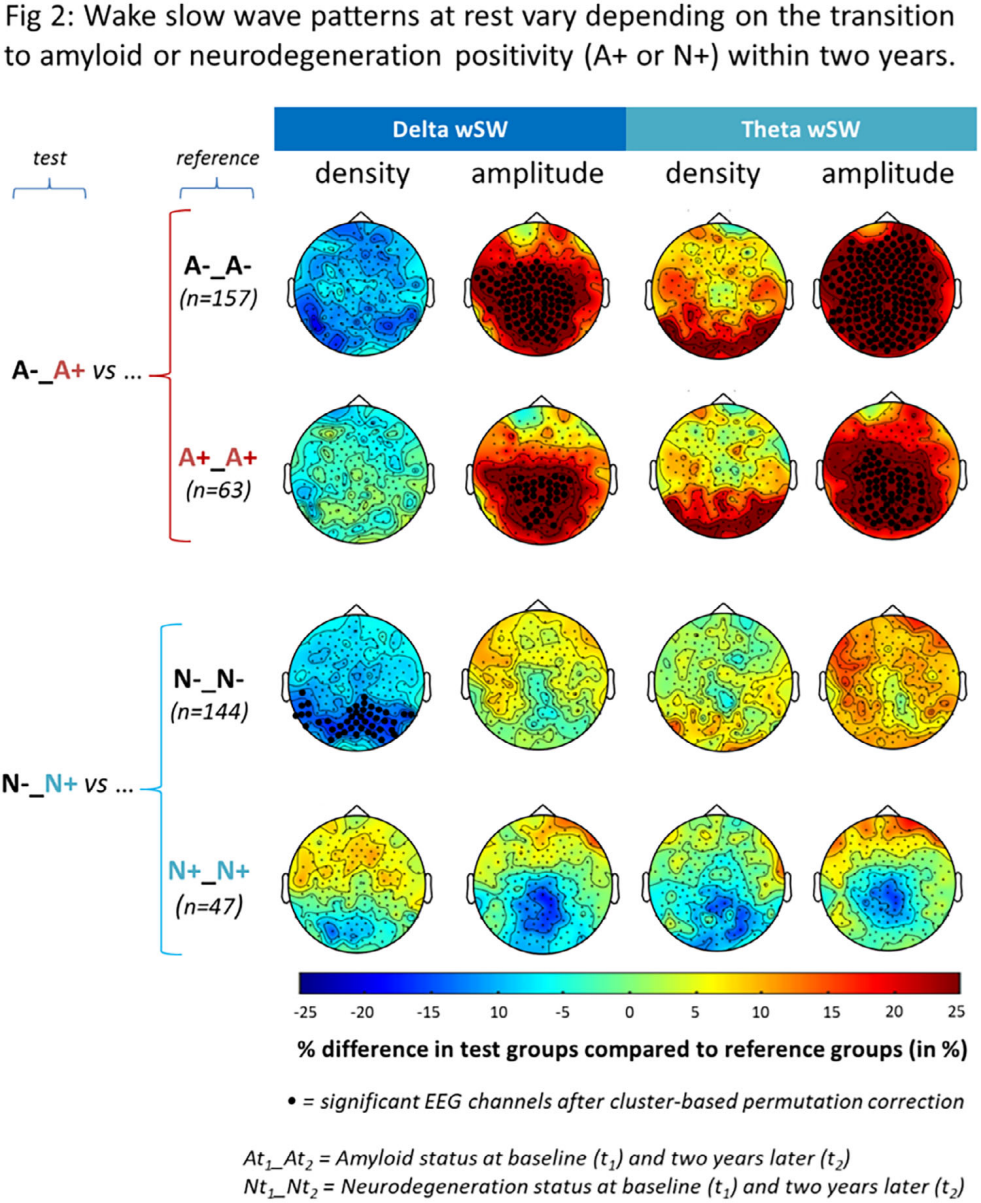# Wake slow waves during resting‐state EEG vary with current and two‐year changes in amyloid and neurodegeneration status in older adults with memory complaints

**DOI:** 10.1002/alz70856_101063

**Published:** 2025-12-25

**Authors:** Pierre Champetier, Claudia Albero, Filipa Raposo Pereira, Rubén Herzog, Maximilien Chaumon, Marion Houot, Nathalie George, Isabelle Arnulf, Delphine Oudiette, Thomas Andrillon, INSIGHT‐preAD group

**Affiliations:** ^1^ Sorbonne Université, Paris Brain Institute (ICM), INSERM, CNRS, UMR‐1127, DreamTeam, Paris, France; ^2^ AP‐HP, Hôpital Pitié‐Salpêtrière, Service des Pathologies du Sommeil, National Reference Center for Narcolepsy, Paris, France; ^3^ Sorbonne Université, Paris Brain Institute (ICM), INSERM, CNRS, CENIR MEG‐EEG, Paris, France; ^4^ Institut de la Mémoire et de la Maladie d’Alzheimer (IM2A), Départment de Neurologie, Paris, France

## Abstract

**Background:**

An increasing body of evidence has underscored the critical role of sleep slow waves in facilitating the clearance of brain waste, affecting the accumulation of Alzheimer's disease (AD) brain lesions. Recent studies have identified sleep‐like slow waves in wake EEG of young adults, often interpreted as intrusions of local sleep during wakefulness. The occurrence of these wake slow waves (wSW) is known to increase during sleep deprivation, and can predict behavioral errors across various tasks. This study aimed to compare wSW features based on 1) amyloid (A‐/A+) and neurodegeneration (*N*‐/N+) status, and 2) transitions to amyloid or neurodegeneration positivity (A+ or N+).

**Method:**

We included 314 elderly individuals with memory complaints from the INSIGHT‐preAD cohort (76.5 ± 3.5 years, range: 70‐85 years). Amyloid (A) and neurodegeneration (N) status were assessed at baseline and 2 years later using Florbetapir and ^18^F‐FDG PET scans. wSW features (density and amplitude within delta and theta frequency bands) were extracted from 2 minutes of resting‐state high‐density EEG recordings (256 electrodes). ANOVA tests were performed on each EEG channel to compare wSW features based on 1) A and N status, and 2) transitions to A+ or N+ between the two sessions. All analyses were adjusted for age, sex, ApoE genotype and education level. Multiple comparisons were corrected using a cluster‐based permutation approach.

**Result:**

Compared to A‐N‐ participants, the other groups (A+N‐, *n* = 45; A‐N+, *n* = 44; and A+N+, *n* = 37) exhibited significantly lower delta wSW density. Moreover, A+N+ individuals showed significantly higher wSW amplitude in both delta and theta bands.

Participants transitioning from A‐ to A+ over 2 years (*n* = 16) had significantly higher wSW amplitude (delta and theta) than those remaining A‐, as well as compared to stable A+ individuals. Participants transitioning from N‐ to N+ (*n* = 33) exhibited significantly lower delta wSW density compared to those remaining N‐.

**Conclusion:**

Our findings suggest that wSW features may serve as early EEG biomarkers for amyloid deposition and neurodegeneration in AD. These metrics, measurable during a simple and brief resting‐state EEG, show potential for identifying at‐risk individuals at very early stages of the disease, even before cognitive decline onset.